# Evaluating the impact of metabolic syndrome on aging in US adults: a cross-sectional study from NHANES

**DOI:** 10.3389/fpubh.2025.1593214

**Published:** 2025-06-02

**Authors:** Linke Li, Senlin Wang, Hong Yang, Jiang Xie, Mengyuan Wang

**Affiliations:** ^1^The Center of Obesity and Metabolic Diseases, Department of General Surgery, Affiliated Hospital of Southwest Jiaotong University, The Third People's Hospital of Chengdu, Chengdu, China; ^2^College of Medicine, Southwest Jiaotong University, Chengdu, China; ^3^Department of Aesthetic Medicine, The Third People's Hospital of Chengdu (Affiliated Hospital of Southwest Jiaotong University), College of Medicine, Southwest Jiaotong University, Chengdu, Sichuan, China

**Keywords:** MetS, aging, NHANES, cross-sectional study, PhenoAge

## Abstract

**Objective:**

As the global population ages, promoting healthy aging has become increasingly important. Although metabolic syndrome (MetS) is associated with aging, current research evidence remains insufficient.

**Methods:**

Data from the 1999–2010 National Health and Nutrition Examination Survey (NHANES) were used for analysis. This dataset includes comprehensive demographic characteristics and biochemical markers. Weighted multivariable logistic regression models were employed to analyze the associations between MetS, its components, and accelerated aging (quantified by PhenoAge Acceleration, PhenoAgeAccel). Restricted cubic spline (RCS) curves were used to explore non-linear relationships between MetS, its components, and PhenoAgeAccel.

**Results:**

The study included 10,049 participants, with a mean age of 45.90 years. Compared to participants without MetS, those with MetS showed an increase in age by 0.61 years (β 0.61, 95% CI 0.12–1.10). Among the five components of MetS, after adjusting for all covariates, significant positive associations were observed only for hypertension (β 0.92, 95% CI 0.36–1.48), reduced HDL-C (β 0.66, 95% CI 0.28–1.04), and elevated blood glucose (β 1.43, 95% CI 0.92–1.94).

**Conclusion:**

Our study demonstrates that patients with MetS are associated with an increased risk of biological aging, with significant contributions from hypertension, elevated blood glucose levels, and reduced HDL-C to the aging process. These findings provide valuable insights for developing public health strategies to mitigate aging.

## 1 Introduction

It is projected that by 2050, the proportion of the global population aged 60 and above will reach 22% (1). Concurrently, aging is often accompanied by numerous chronic diseases, including cardiovascular diseases, respiratory diseases, cancer, and type II diabetes, which impose a significant economic burden on families and society (2). Consequently, monitoring aging has been a focal point of scientific research. Up until now, various biomarkers have been proposed to collectively define the aging phenotype, including genomic instability, telomere shortening, epigenetic changes, and proteostasis loss (3–5). Evidence suggests that patients with MetS exhibit significantly shorter telomere lengths (TL), however current evidence is limited and does not establish a critical TL threshold for defining biological age (6). Recently, Phenotypic age (PhenoAge) has been proposed as a measure to reflect an individual's biological aging status. Compared to relatively complex and expensive tests like those for genomic instability and DNA methylation, the calculation of PhenoAge typically relies on routine clinical laboratory markers, such as blood chemistry and inflammatory markers. By integrating multiple biomarkers, PhenoAge provides more accurate information on health and aging than chronological age, making it a more practical and cost-effective tool for assessing biological age.

MetS is characterized by a group of interconnected conditions, including obesity, hypertension, elevated triglyceride levels, and insulin resistance (7). Although MetS itself is not an absolute risk indicator, it remains a significant risk factor for cardiovascular diseases, diabetes, chronic kidney disease, hyperinsulinemia, and various mental disorders (8). Research indicates that aging-related markers, such as telomerase, are present at increased levels in individuals with MetS (9). Nannini et al. reported that accelerated intrinsic epigenetic age significantly increases the likelihood of developing MetS (6). Aging and MetS, potentially due to shared risk factors such as unhealthy diet, obesity, and various underlying chronic conditions (10). In this context, integrating MetS and aging as a whole does not facilitate disease management and risk prevention. Despite some evidence highlighting the association between MetS and aging, there is currently a lack of large-scale epidemiological studies to clearly define the contribution of MetS to accelerated aging. Measuring the PhenoAge of MetS patients can help identify and stratify the individuals at highest risk for accelerated aging (11). Additionally, there is no clear epidemiological evidence to evaluate whether each component of MetS is positively correlated with aging. To address these knowledge gaps, we aim to answer these questions by analyzing NHANES data and further investigating the relationship between MetS, its components, and aging in the general U.S. population.

## 2 Methods

### 2.1 Study design and population

**Figure 1:**
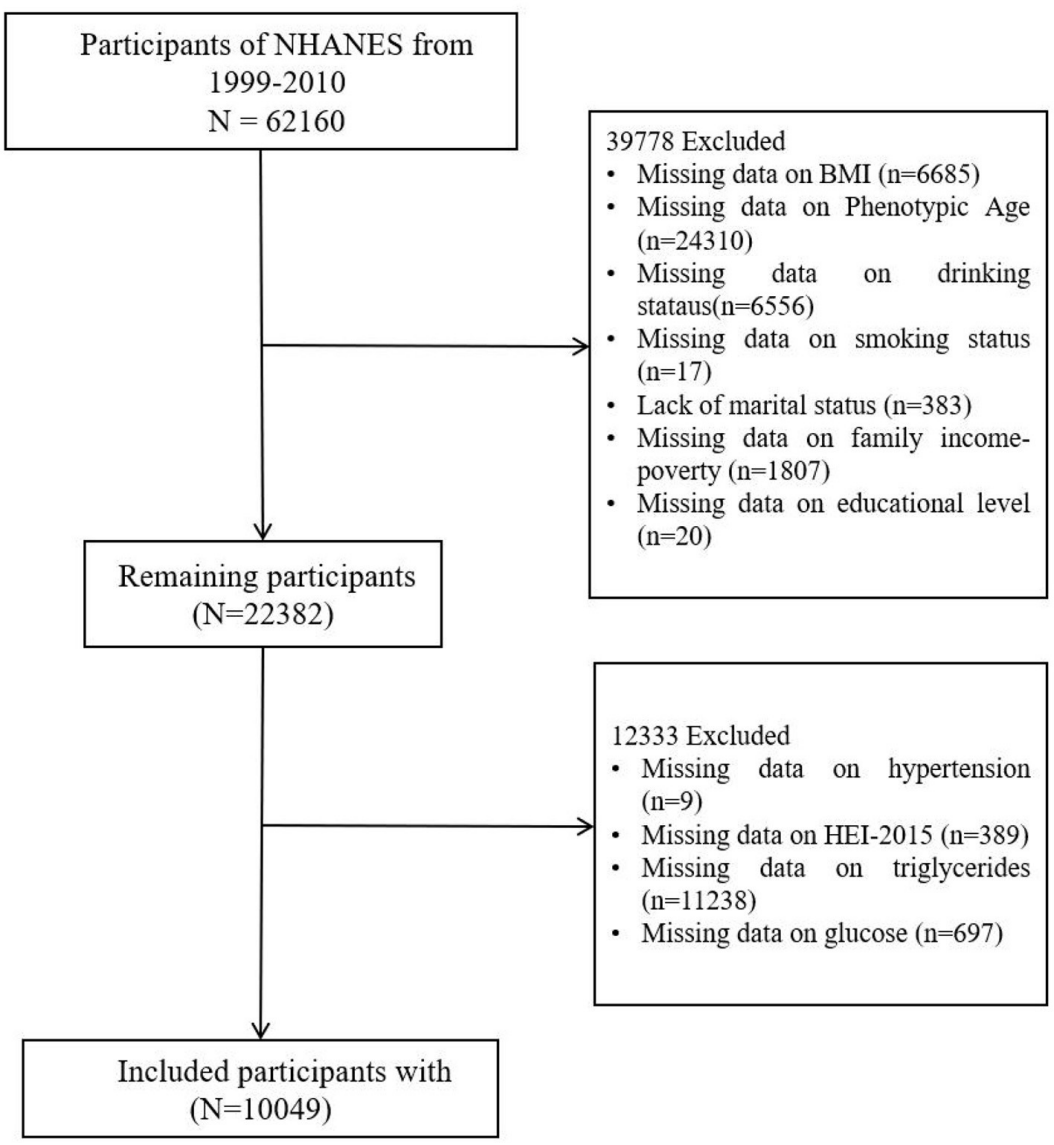
Flow chart of the patients included in the study.

NHANES provides a comprehensive, ongoing assessment of the health and nutritional status of the U.S. population through a combination of interviews, physical examinations, and laboratory tests. NHANES follows stringent ethical protocols, including obtaining informed consent from all participants and ensuring the confidentiality and privacy of their data. The study protocol is reviewed and approved by the National Center for Health Statistics Research Ethics Review Board. In this study, we included participants from the 1999–2010 NHANES cohorts (Figure 1). After handling missing data for the necessary variables (by deletion), a total of 10,049 participants were included in the final analysis.

### 2.2 Assessment of PhenoAge and mets

PhenoAge, a well-established marker, is utilized to assess the biological aging process through extensive research (12–14). It is computed based on 10 indicators, including chronological age, albumin, creatinine, glucose, C-reactive protein, lymphocyte percentage, mean cell volume, red cell distribution width, alkaline phosphatase, and white blood cell count (14). The incorporation of these biological markers, intimately associated with the functionality and metabolism of various bodily systems, allows for a more accurate prediction of health-related outcomes (15). PhenoAgeAccel is defined as the residual obtained by subtracting the influence of chronological age using a linear regression model (16). Specifically, PhenoAgeAccel was derived as the residual from a linear regression model in which PhenoAge was regressed on chronological age: (PhenoAgeAccel = PhenoAge – PhenoAge∧), where PhenoAge∧ represents the predicted phenotypic age based on chronological age. This method has been previously validated in NHANES populations. Smaller residual values indicate a slower biological aging process (17). PhenoAgeAccel aids in understanding the contrast between physiological aging pace and chronological age.

The National Cholesterol Education Program's Adult Treatment Panel III (NCEP ATP III) in 2005 for diagnosing MetS. The criteria include a waist circumference of ≥102 cm for men or ≥88 cm for women, HDL-C levels < 40 mg/dL for men or < 50 mg/dL for women, triglycerides ≥1.7 mmol/L, blood pressure ≥130/85 mmHg or use of antihypertensive medication, and fasting glucose ≥5.6 mmol/L or use of antidiabetic medication (18, 19).

### 2.3 Assessment of covariates

Hypertension is defined as a systolic blood pressure ≥140 mmHg, diastolic blood pressure ≥90 mmHg, self-reported physician diagnosis, or current use of antihypertensive medic. Diagnostic criteria for diabetes include a glycated hemoglobin (HbA1c) level ≥6.5%, fasting blood glucose level greater than or equal to 7 mmol/L, oral glucose tolerance test (OGTT), self-reported diabetes, or current use of antidiabetic medications such as metformin (20, 21). Covariates were defined as smoking status (current: serum cotinine ≥10 ng/mL or self-reported daily smoking; former: quit >1 year; never), alcohol consumption (current: ≥1 drink/month; former; never), central obesity (waist circumference ≥102 cm for men or ≥88 cm for women), and HDL-C levels (< 40 mg/dL for men or < 50 mg/dL for women). Marital status, level of education, race, Body Mass Index (BMI), household income level [Poverty Income Ratio (PIR) < 1.3, 1.3–3.5, >3.5], smoking, hypertension (22), and alcohol consumption are also considered important covariates (23, 24) HEI-2015 (Healthy Eating Index-2015) is a scoring system designed to assess the overall quality of diet based on adherence to the 2015–2020 Dietary Guidelines for Americans (25–27).

### 2.4 Statistical analysis

All analyses incorporated NHANES examination weights, accounting for primary sampling units (PSUs), strata, and individual-level weights, with variance estimation adjusted for PSU clustering using Taylor linearization (28). Variance inflation factors (VIF) confirmed no severe multicollinearity (all VIF < 5) among MetS components. Baseline characteristics were presented as weighted mean ± standard error (SE) for continuous variables and unweighted counts with weighted percentages for categorical variables. Weighted linear regression models calculated regression coefficients (β) and 95% confidence intervals (CIs). Scatter plots, unweighted, visualized the distribution of MetS components (blood glucose, waist circumference, triglycerides, HDL-C) against PhenoAge. RCS models explored potential non-linear relationships between these components and PhenoAge, excluding hypertension status. Key thresholds identified included HDL-C >50 mg/dL, which was associated with reduced PhenoAgeAccel (β = −0.32, 95% CI: −0.55 to −0.09), and fasting glucose >100 mg/dL (5.6 mmol/L), which was linked to an increased risk of accelerated aging (β = 0.41 per 10 mg/dL increase, 95% CI: 0.28–0.54). These findings suggest the clinical importance of managing HDL-C levels above 50 mg/dL and keeping fasting glucose levels below 100 mg/dL to mitigate accelerated aging risks. Sensitivity analyses were conducted as follows. Firstly, α-Klotho (klotho) is a protein involved in suppressing oxidative stress and inflammation. It has been reported as the basis for many aging phenotypes and longevity in animal models (29). Serum Klotho was measured only by the NHANES working group during 2007 to 2010, thus 2,228 out of 10,049 participants underwent serum Klotho testing. We validated our findings by incorporating the measured Klotho values as exposure variables in regression analyses with PhenoAge as the outcome. Secondly, the International Diabetes Federation (IDF) established diagnostic criteria for MetS in 2009 (8). Based on the IDF 2009 criteria, which account for racial differences and central obesity, they are more applicable globally. A second sensitivity analysis validated findings by redefining MetS as exposure variables in regression analyses with PhenoAge and accelerated PhenoAge as outcomes. Finally, regarding the insightful question concerning potential cohort effects arising from the extended data time span, we addressed this potential bias by incorporating the survey years as categorical covariates in the statistical model (30). Subgroup analyses were conducted by gender, age, BMI, race, PIR, smoking, and alcohol history, with interactions tested using the likelihood ratio test. Analyses were performed in R (version 4.1.3), with significance at *P* < 0.05. No formal adjustments were made for multiple comparisons. Findings from secondary and sensitivity analyses should be interpreted with caution as exploratory results (31).

## 3 Results

### 3.1 Baseline characteristics

The study included a total of 10,049 participants with an average age of 45.90 years (SE 0.35), predominantly male (50.98%). Among all participants, the majority were Non-Hispanic White. Additionally, 63.29% were married or living with a partner, 51.27% had never smoked, and only 13.04% had never consumed alcohol. Participants with MetS had higher BMI, older PhenoAge, and more comorbidities compared to those without the syndrome (Table 1).

**Table 1:** Characteristics of participants in NHANES 1999–2010 (weighted).

**Characteristics**	**Metabolic syndrome**			
	**Overall**	**No**	**Yes**	***P*** **-values**
**Age, mean (SE), y**	45.90 (0.35)	42.74 (0.39)	53.36 (0.40)	< 0.0001
**Sex**, ***n*** **(%)**				0.51
Female	4,926 (49.02)	3,132 (49.96)	1,794 (50.87)	
Male	5,123 (50.98)	3,438 (50.04)	1,685 (49.13)	
**BMI, mean (SE), kg/m** ^**2**^	28.25 (0.11)	26.45 (0.08)	32.51 (0.20)	< 0.0001
**PhenoAgeAccel**	−0.72 (0.11)	−1.79 (0.11)	1.80 (0.22)	< 0.0001
**Phynotypicage**	41.05 (0.39)	36.65 (0.39)	51.42 (0.46)	< 0.0001
**HEI-2015 score, mean (SE)**	49.66 (0.30)	49.75 (0.36)	49.44 (0.34)	0.44
**HDL-C, mg/dL**	52.48 (0.28)	55.94 (0.30)	44.32 (0.33)	< 0.0001
**Triglycerides, mean (SE), mmol/L**	1.58 (0.02)	1.26 (0.01)	2.34 (0.05)	< 0.0001
**Waist circumference, mean (SE), cm**	96.95 (0.27)	91.82 (0.24)	109.07 (0.42)	< 0.0001
**Blood glucose, mean (SE), mmol.L**	5.39 (0.02)	5.06 (0.02)	6.19 (0.05)	< 0.0001
**Alkaline phosphatase, mean (SE), u.L**	70.04 (0.54)	67.88 (0.56)	75.16 (0.86)	< 0.0001
**Albumin, mean (SE), g.L**	42.97 (0.08)	43.31 (0.09)	42.15 (0.09)	< 0.0001
**Creatinine, mean (SE), umol.L**	76.59 (0.55)	75.02 (0.42)	80.31 (1.20)	< 0.0001
**C reactive protein, mean (SE), mg.dl**	0.41 (0.01)	0.34 (0.01)	0.57 (0.02)	< 0.0001
**WBC, mean (SE), 1,000cells.ul**	6.77 (0.03)	6.57 (0.03)	7.24 (0.04)	< 0.0001
**Lymphocyte, mean (SE), %**	30.01 (0.14)	30.22 (0.16)	29.52 (0.20)	0.003
**Mean cell volume, mean (SE), femtoliters**	90.08 (0.11)	90.33 (0.12)	89.49 (0.13)	< 0.0001
**Red cell distribution width, mean (SE)**	12.66 (0.02)	12.59 (0.02)	12.82 (0.03)	< 0.0001
**Race**, ***n*** **(%)**				0.01
Mexican American	1,998 (19.88)	1,250 (7.00)	748 (7.01)	
Non-Hispanic Black	1,791 (17.82)	1,239 (10.36)	552 (8.91)	
Non-Hispanic White	5,275 (52.49)	3,387 (72.49)	1,888 (75.94)	
Other	985 (9.8)	694 (10.15)	291 (8.14)	
**Educational level**, ***n*** **(%)**				< 0.0001
9-12th Grade or below	2,823 (28.09)	1,662 (16.54)	1,161 (22.55)	
College graduate or above	2,032 (20.22)	1,533 (28.52)	499 (17.45)	
High school Grad/GED or equivalent	2,407 (23.95)	1,499 (24.24)	908 (29.86)	
Some college or AA degree	2,787 (27.73)	1,876 (30.71)	911 (30.14)	
**Martial status**, ***n*** **(%)**				< 0.0001
Widowed/divorced/separated	2,175 (21.64)	1,215 (14.69)	960 (22.35)	
Married/living with partner	6,360 (63.29)	4,136 (66.27)	2,224 (68.41)	
Never married	1,514 (15.07)	1,219 (19.04)	295 (9.23)	
**Smoking status**, ***n*** **(%)**				< 0.0001
Former	2,732 (27.19)	1,563 (22.95)	1,169 (32.39)	
Never	5,152 (51.27)	3,508 (51.99)	1,644 (46.73)	
Now	2,165 (21.54)	1,499 (25.05)	666 (20.88)	
**PIR**, ***n*** **(%)**				< 0.0001
< 1.3	2,727 (27.14)	1,647 (17.54)	1,080 (22.21)	
1.3-3.5	3,944 (39.25)	2,568 (36.02)	1,376 (37.36)	
≥3.5	3,378 (33.62)	2,355 (46.44)	1,023 (40.43)	
**Hypertension**, ***n*** **(%)**				< 0.0001
No	5,798 (57.7)	4,698 (76.89)	1,100 (35.86)	
Yes	4,251 (42.3)	1,872 (23.11)	2,379 (64.14)	
**Diabetes status**, ***n*** **(%)**				< 0.0001
DM	1,660 (16.52)	399 (3.86)	1,261 (29.39)	
IFG	887 (8.83)	324 (3.62)	563 (15.94)	
IGT	583 (5.80)	356 (3.49)	227 (5.14)	
No	6,919 (68.85)	5,491 (89.04)	1,428 (49.53)	
**Drinking status**, ***n*** **(%)**				< 0.0001
Former	2010 (20.00)	1102 (13.54)	908 (23.34)	
Never	1310 (13.04)	774 (9.95)	536 (13.27)	
Now	6729 (66.96)	4694 (76.51)	2035 (63.40)	

BMI, body mass index; HDL-C, High-Density Lipoprotein Cholesterol; HEI, Healthy Eating Index−2015; IFG, impaired fasting glucose; IGT, impaired glucose golerance; WBC, White Blood Cells.

For continuous variables, *P*-values were calculated using a weighted *Student's* t-test, and for categorical variables, *P*-values were computed using weighted chi-square tests.

### 3.2 Association between mets and its components with PhenoAge/PhenoAgeAccel

**Figure 2:**
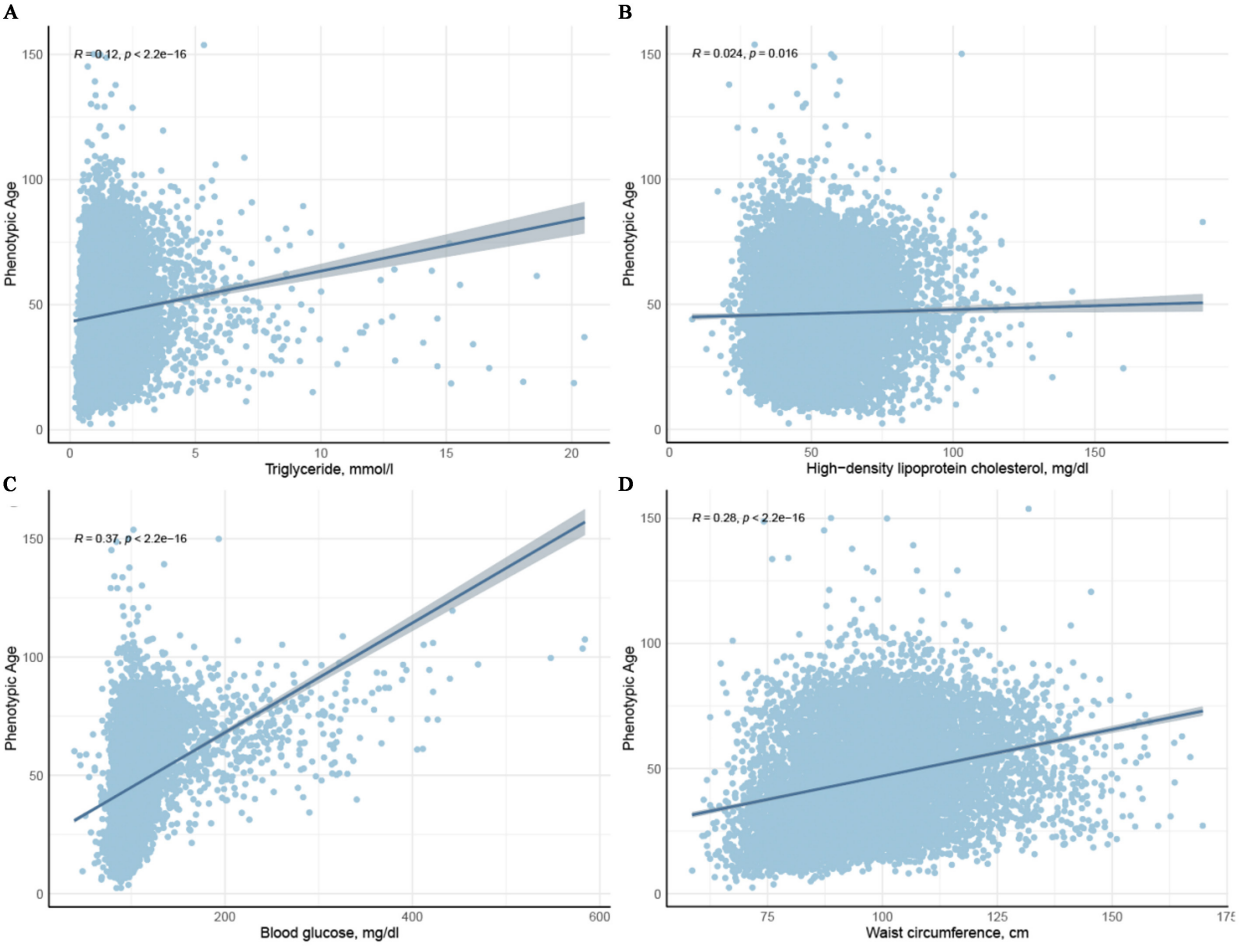
Scatter plots depicting the relationship between the four components of metabolic syndrome and Phenotypic age.

Scatter plots illustrating the associations between PhenoAge and each component of MetS (except hypertension) are shown in Figure 2. It is evident that triglycerides, blood glucose, waist circumference, and HDL-C are positively correlated with PhenoAge (*R*^2^ > 0, *P* < 0.05). Further weighted linear regression analysis (Table 2) reveals that participants with MetS age an additional 0.61 years compared to those without the syndrome (β 0.61, 95% CI 0.12–1.10). Among the five components of MetS, after adjusting for all covariates, significant positive correlations were observed only for hypertension (β 0.92, 95% CI 0.36–1.48), reduced HDL-C (β 0.66, 95% CI 0.28–1.04), and raised blood glucose (β 1.43, 95% CI 0.92–1.94). Though triglycerides (TG) and waist circumference (WC) did not reach significance, their effect sizes (TG: β = 0.15; WC: β = 0.20) aligned with known metabolic aging pathways, possibly masked by collinearity (VIF < 5) or diagnostic thresholds. Supplementary Table 1 presents the weighted linear regression results for MetS and its components with PhenoAgeAccel, consistent with the associations observed for PhenoAge.

**Table 2:** The associations of metabolic syndrome with PhenoAge.

**Characteristic**	**Crude model**		**Model 1**		**Model 2**	
	β **(95 % CI)**	***P***	β **(95 % CI)**	***P***	β **(95 % CI)**	***P***
**Metabolic syndrome**					
No	ref		ref		ref	
Yes	14.76 (13.81, 15.72)^a^	< 0.0001	2.49 (2.03,2.95)^a^	< 0.0001	0.61 (0.12, 1.10)^a^	0.01
**Hypertension**					
No	ref		ref		ref	
Yes	19.2 (18.39, 20.00)^a^	< 0.0001	1.58 (1.01, 2.15)^a^	< 0.0001	0.92 (0.36, 1.48)^a^	0.002
**Raised triglyceride**					
No	ref		ref		ref	
Yes	6.56 (5.50, 7.63)^a^	< 0.0001	0.94 (0.45, 1.43)^a^	< 0.001	0.15 (−0.29, 0.60)	0.49
**Reduced HDL-C**					
No	ref		ref		ref	
Yes	0.20 (−0.72, 1.12)^a^	0.67	1.56 (1.09, 2.02)^a^	< 0.0001	0.66 (0.28, 1.04)^a^	< 0.001
**Central obesity**					
No	ref		ref		ref	
Yes	10.61 (9.71, 11.51)^a^	< 0.0001	0.65 (0.24, 1.06)^a^	0.002	0.20 (−0.17, 0.57)	0.29
**Raised blood glucose**					
No	ref		ref		ref	
Yes	15.54 (14.60, 16.48)^a^	< 0.0001	3.14 (2.71, 3.57)^a^	< 0.0001	1.43 (0.92, 1.94)^a^	< 0.0001

CI, confidence interval; Crude model, unadjusted; Model 1, adjusted for age, sex, race, BMI; Model 2, adjusted for variables in Model 1 plus, marital status, drinking status, smoking status, educational level, hypertension, diabetes, PIR, and HEI-2015.

^a^Statistically significant.

### 3.3 RCS curve and sensitivity analysis

**Figure 3:**
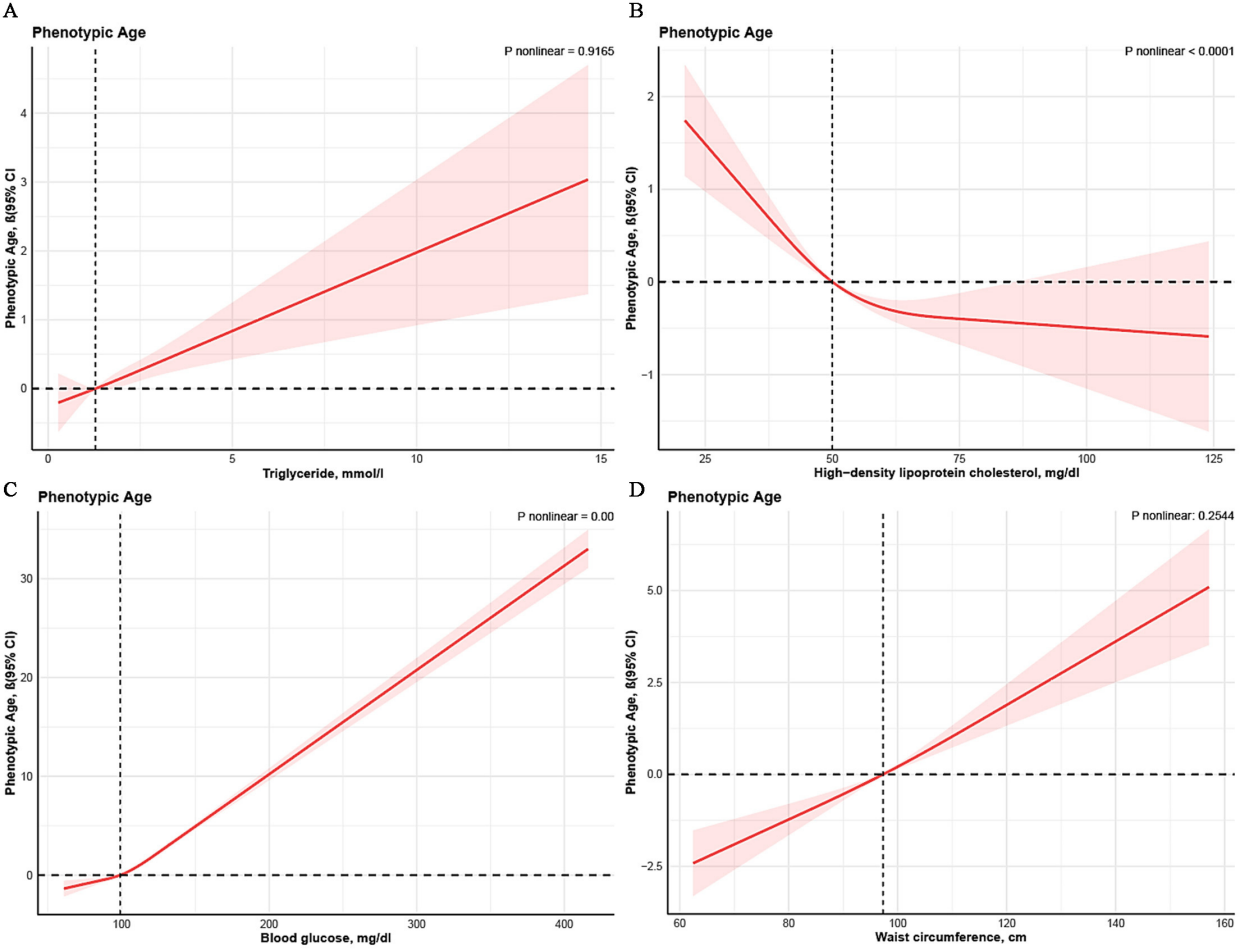
Non-linear association between metabolic syndrome and PhenoAge. Cubic spline models adjusted for age, sex, race, BMI, marital status, drinking status, smoking status, educational level, hypertension, diabetes, PIR, and HEI-2015. Knots = 3. CI, confidence interval.

Non-linear associations between four principal components of MetS and PhenoAge are depicted in Figure 3 through smoothed visualization techniques. The results revealed non-linear relationships for HDL-C and blood glucose with PhenoAge (*P* for non-linearity < 0.05). Specifically, a turning point was observed for HDL-C when levels exceeded 50 mg/dL, although the confidence intervals were relatively wide. For blood glucose, there was a marked positive correlation with PhenoAge beyond 100 mg/dL. Similar patterns were observed for triglycerides and waist circumference, indicating a significant association with increased PhenoAge.

Supplementary analyses provide additional insights into how MetS and its components influence serum α-Klotho levels, further supporting the robustness of our findings. These results are detailed in Supplementary Tables 2, 3. The study revealed a significant negative correlation between participants with MetS and serum α-Klotho levels. Given the inverse relationship between serum α-Klotho levels and accelerated aging, this sensitivity analysis further corroborated our findings. The results of sensitivity analyses examining the associations between MetS and its components—defined according to the IDF-2009 criteria—and both PhenoAge and PhenoAge Acceleration are presented in Supplementary Tables 4, 5. Consistent with the findings using the ATP III definition of MetS, adopting the IDF-2009 criteria resulted in stronger associations between MetS and PhenoAge (β 0.64, 95% CI 0.18–1.10) or PhenoAgeAccel (β 0.75, 95% CI 0.27–1.24). The Supplementary Tables 6, 7 demonstrate that after incorporating survey cycles as covariates, the associations between metabolic syndrome (and its components) with PhenoAge and PhenoAge acceleration remained consistent with the primary analyses.

### 3.4 Subgroup analysis

Table 3 presents the results of subgroup analyses to assess heterogeneity across different populations or disease states. After adjusting for all covariates, we did not observe heterogeneity in gender, BMI, race, smoking status, and drinking status. The results suggest that within the age groups, participants younger than 65 years showed a more significant impact of MetS status on PhenoAge (β 3.29, 95% CI 2.40 to 4.18), whereas no such variation was observed in the group aged 65 years or older. In the PIR subgroup analysis, the overall trend indicated that higher income is associated with a lower risk of biological aging in the presence of MetS. The β values for PIR < 1.3, 1.3–3.5, and >3.5 were 2.84, 2.83, and 2.21, respectively, all *P* < 0.05, with *P* for interaction = 0.03.

**Table 3:** The associations of metabolic syndrome with PhenoAge in subgroups.

**Characteristic**	**Metabolic syndrome**			
	**No**	**Yes**, β **(95 % CI)**^a^	***P***	***P*** **for interaction**
**Sex**				0.67
Male	ref	2.47 (1.42, 3.52)	< 0.0001	
Female	ref	2.87 (1.97, 3.77)	< 0.0001	
**Age group**				< 0.0001
< 65	ref	3.29 (2.40, 4.18)	< 0.0001	
>=65	ref	0.43 (−0.61, 1.46)	0.41	
**BMI group**				0.27
Overweight	ref	2.89 (1.69, 4.09)	< 0.0001	
Obese	ref	3.07 (2.00, 4.14)	< 0.0001	
Normal	ref	2.01 (−0.44, 4.46)	0.11	
**Race**				0.63
Non-Hispanic White	ref	2.72 (1.74, 3.71)	< 0.0001	
Non-Hispanic Black	ref	1.94 (0.08, 3.79)	0.04	
Mexican American	ref	2.5 (1.28, 3.71)	< 0.001	
Other	ref	3.08 (1.09, 5.07)	0.003	
**PIR**				0.03
< 1.3	ref	2.84 (1.06, 4.61)	0.002	
1.3–3.5	ref	2.83 (1.76, 3.91)	< 0.0001	
≥3.5	ref	2.21 (1.03, 3.38)	< 0.001	
**Smoking status**				0.05
Former	ref	2.19 (0.97, 3.40)	< 0.001	
Never	ref	2.58 (1.47, 3.69)	< 0.0001	
Now	ref	3.69 (1.56, 5.81)	< 0.001	
**Drinking status**				0.15
Former	ref	4.24 (2.61, 5.87)	< 0.0001	
Heavy	ref	3.86 (2.15, 5.57)	< 0.0001	
Moderate	ref	2.63 (0.64, 4.62)	0.01	
Mild	ref	1.19 (−0.09, 2.46)	0.07	
Never	ref	1.57 (−0.78, 3.93)	0.19	

BMI, body mass index; CI, confidence interval; DM, diabetes; PreDM (IFG, impaired fasting glucose; IGT, impaired glucose golerance).

^a^Model adjusted for age, sex, race, BMI, marital status, drinking status, smoking status, educational level, hypertension, diabetes, PIR, and HEI-2015.

Supplementary Table 8 displays the associations of MetS with PhenoAgeAccel in subgroups. Heterogeneity was only observed within the race subgroup. The results indicate that participants from the Non-Hispanic White group (β = 0.96, 95% CI 0.38 to 1.54) are more susceptible to significant effects of MetS on PhenoAgeAccel, whereas this phenomenon was not observed in the Non-Hispanic Black, Mexican American, and other groups.

## 4 Discussion

This large-scale study aimed to evaluate the impact of MetS on participants' biological aging and accelerated aging. The study revealed that participants with MetS experienced a 0.61-year increase in the PhenoAge of aging. RCS analysis unveiled non-linear associations between HDL-C, blood glucose, and PhenoAge. Sensitivity analysis further corroborated the robustness and reliability of our findings. Subgroup analysis indicated that participants under the age of 65, and low PIR levels exhibited a more significant influence of MetS on PhenoAge.

Previous studies have shown that epigenetic age acceleration is positively correlated with the severity score of MetS and the number of MetS components (6). Our study further confirms that similar trends are observed with PhenoAge and PhenoAge acceleration. PhenoAge offers more advantages in health management and disease prevention than chronological age, as it better represents an individual's aging status and is more accessible in routine clinical practice, making it a more practical and cost-effective tool for assessing biological age (32).

Aging is accompanied by molecular damage and a decline in maintenance and repair mechanisms, particularly oxidative damage induced by reactive oxygen species (ROS), which significantly impacts cellular homeostasis and physiological functions (33). ROS-induced damage includes harm to mitochondrial DNA and disruption of the electron transport chain, further increasing ROS production and ultimately leading to progressive cellular dysfunction and death (34). Among various aging assessment tools, PhenoAge and epigenetic clocks (such as DNA methylation-based biomarkers) together form a multidimensional aging evaluation framework (35). Crucially, PhenoAge—as a core clinical biomarker-based tool—differs fundamentally from epigenetic clocks: while epigenetic clocks strongly correlate with age-related health outcomes (e.g., frailty and cognitive decline), PhenoAge's broad coverage of clinical indicators grants it unique advantages in holistic aging assessment. Future studies should prioritize direct comparisons between PhenoAge and epigenetic clocks to better contextualize these findings within a broader aging research framework. Most importantly, investigating how PhenoAge correlates with functional outcomes (e.g., frailty and cognitive decline) will not only enhance its clinical utility but also provide PhenoAge-driven insights into how MetS influences aging at both molecular and functional levels.

Hyperglycemia-induced ROS and PKC can also activate NF-κB in mesangial cells, participating in immune and inflammatory responses (36). Chronic low-grade systemic inflammation is a hallmark of aging. Senescent cells often secrete an inflammatory mixture of cytokines, chemokines, and matrix metalloproteinases, which can cause dysfunction in insulin signaling pathways (37). The mechanisms by which MetS accelerates aging are related to obesity, with oxidative stress and inflammation serving as critical links. However, we did not observe evidence of biological aging and accelerated aging in the MetS components Raised triglycerides and Central obesity. According to the MetS definition of Raised triglycerides and Central obesity, we believe this is partly due to our study not excluding participants currently using lipid-lowering medications and the racial heterogeneity in the definition of Central obesity (38).

The non-significant associations of triglycerides and waist circumference deserve nuanced interpretation. First, the ATP-III criteria's fixed thresholds (waist circumference ≥102/88 cm) may not capture ethnic-specific risks, as our Mexican American subgroup had mean 96.95 cm. Second, lipid-lowering medications (not excluded in primary analysis) could attenuate triglyceride effects. Most importantly, their linear dose-response relationships with PhenoAge in RCS analyses (Figure 3) suggest cumulative harm below diagnostic cutoffs, echoing studies linking visceral adiposity to inflammaging and hypertriglyceridemia to mitochondrial dysfunction.

In this study, we explored the potential heterogeneity in the relationship between MetS and biological aging, taking into account the role of sex and race/ethnicity as potential effect modifiers. Although the interaction between sex and MetS did not reach statistical significance (*P* = 0.67), the data suggest that sex may play a role in moderating the impact of MetS on aging. Specifically, MetS was significantly associated with increased PhenoAge in both men and women, but the association appeared to be stronger in women (β = 2.87, *P* < 0.0001). This stronger effect in women may be attributed to hormonal changes post-menopause, which influence fat distribution, insulin sensitivity, and lipid metabolism, thereby exacerbating MetS and accelerating biological aging. Women also tend to have more pronounced abdominal obesity, a key component of MetS, which may further contribute to the observed sex differences.

Regarding racial/ethnic differences, while we did not find significant modification effects of race/ethnicity on the MetS-aging relationship (all p for interaction >0.05), there were notable differences in MetS prevalence across racial/ethnic groups, as shown in Table 1 (*P* = 0.01). For instance, Non-Hispanic Whites had a higher prevalence of MetS compared to Mexican Americans and Non-Hispanic Blacks. These differences highlight the complex interplay between race, genetic susceptibility, lifestyle factors, and access to healthcare, which may influence the relationship between MetS and biological aging. However, due to the limited sample size in certain subgroups, further stratified analyses were not conducted in this study. This remains an important area for future research, where larger and more racially/ethnically balanced studies are needed to comprehensively examine potential effect modification.

Both sex and race/ethnicity may therefore be important modifiers of the relationship between MetS and biological aging. Future studies should continue to explore these factors to provide more nuanced insights into how different populations experience the effects of MetS on aging.

In the RCS analysis, there is evidence of a non-linear association between blood glucose levels and HDL-C levels with PhenoAge. The study found that prolonged high blood glucose levels lead to the glycation of red blood cell membranes and hemoglobin, resulting in decreased oxygen-carrying capacity (39). This causes tissues and cells in the body to remain in a state of hypoxia. Additionally, hyperglycemia is often accompanied by insulin deficiency, which results in more protein breakdown than synthesis, leading to hypoproteinemia and negative nitrogen balance (40). Free cholesterol can be endocytosed by cells and, through oxidation reactions with macrophages, release inflammatory factors that contribute to atherosclerosis and other cardiovascular diseases (41), HDL-C assists in excreting cholesterol from the body in the form of bile, thus reducing free cholesterol levels in the blood (42). Therefore, HDL-C reduces the risk of aging primarily by controlling inflammatory responses. Notably, triglycerides and waist circumference show a significant linear dose-response relationship with PhenoAge. This partially reflects that the potential harm of triglycerides and higher waist circumference on biological aging might be underestimated when defined by MetS criteria. Age itself induces changes in human triglyceride metabolism, including elevated plasma triglyceride levels, decreased postprandial plasma TG clearance, reduced lipolysis in adipose tissue, and increased ectopic fat deposition (43). Defining high TG with a fixed threshold may not be suitable for all age groups, so stratifying triglyceride thresholds based on age might be reasonable. As people age, body fat redistributes, leading to an increase in trunk fat (visceral fat) and a decrease in subcutaneous fat (44). Waist circumference measurements cannot distinguish between subcutaneous and visceral fat (45), which may lead to an underestimation of waist circumference in determining health risks associated with MetS and aging.

The α-Klotho protein is more than just an aging marker; it plays a crucial role in overall health and longevity by exerting various physiological effects on multiple tissues and organs. Therefore, we aimed to corroborate our primary findings by analyzing the relationship between MetS and its components with α-Klotho protein. As a cofactor of FGFs, α-Klotho protein can directly interact with FGFR1c, forming a ternary complex with FGF23, α-Klotho, and FGFR1c (46). Knockout mice of the FGF23 gene exhibit hyperphosphatemia and high serum 1,25(OH)2D, presenting a complex phenotype characterized by premature aging features such as thymus and spleen atrophy (47). Studies have also indicated that α-Klotho is associated with various metabolic diseases, and high concentrations of α-Klotho can cut down the risk of diabetes, kidney disease, and cardiovascular diseases (48, 49). Our sensitivity analysis results further confirm the association between MetS with biological aging and accelerated aging. However, this study has the following methodological limitations that warrant particular attention: the measurement of serum Klotho concentrations was restricted to a subgroup of participants from the 2007–2010 period, which may introduce potential selection bias.

Subgroup analysis revealed that participants under 65 years of age or with lower income levels showed a greater impact of MetS status on their PhenoAge compared to older adults. Adverse lifestyle habits among younger populations, such as poor dietary choices (50) and lower educational attainment (51), might make them more susceptible to the effects of MetS. Furthermore, although age is merely a life cycle marker, it is inherently associated with biological aging and comorbid conditions. Age may appear to contribute more to aging than MetS in statistical analyses, but this might not fully capture age's true impact. Low-income individuals are disproportionately affected by MetS, significantly influencing their physiological age, likely due to limited healthcare access, psychological issues like anxiety and depression, and higher rates of unhealthy habits such as smoking, excessive alcohol consumption, and physical inactivity. While our study provides valuable insights, it has limitations. First, the cross-sectional design prevents establishing causality, limiting observations to associations at a single time point. Second, data may be subject to information and recall bias. Additionally, unmeasured confounders, such as disease duration, lifestyle factors, and other variables, could influence the results.

## 5 Conclusion

Patients with MetS are associated with an increased risk of biological aging, with components of MetS such as hypertension, elevated blood glucose levels, and reduced HDL-C contributing significantly to the aging process. Further research is imperative to gain a more comprehensive understanding of the mechanisms through which MetS accelerates aging and to validate these findings in longitudinal studies.

## Data Availability

The original contributions presented in the study are included in the article/Supplementary material, further inquiries can be directed to the corresponding authors.
