# Correction: Evaluating the impact of metabolic syndrome on aging in US adults: a cross-sectional study from NHANES

**DOI:** 10.3389/fpubh.2025.1642406

**Published:** 2025-06-26

**Authors:** Linke Li, Senlin Wang, Hong Yang, Jiang Xie, Mengyuan Wang

**Affiliations:** ^1^The Center of Obesity and Metabolic Diseases, Department of General Surgery, Affiliated Hospital of Southwest Jiaotong University, The Third People's Hospital of Chengdu, Chengdu, China; ^2^College of Medicine, Southwest Jiaotong University, Chengdu, China; ^3^Department of Aesthetic Medicine, The Third People's Hospital of Chengdu (Affiliated Hospital of Southwest Jiaotong University), College of Medicine, Southwest Jiaotong University, Chengdu, Sichuan, China

**Keywords:** MetS, aging, NHANES, cross-sectional study, PhenoAge

In the published article, there was an error in Affiliation section. Affiliation was erroneously given as “1 College of Medicine, Southwest Jiaotong University, Chengdu, China. 2 The Center of Obesity and Metabolic Diseases, Department of General Surgery, Affiliated Hospital of Southwest Jiaotong University, The Third People's Hospital of Chengdu, Chengdu, China.”

The correct affiliation appears below:

“^1^ The Center of Obesity and Metabolic Diseases, Department of General Surgery, Affiliated Hospital of Southwest Jiaotong University, The Third People's Hospital of Chengdu, Chengdu, China.

“^2^ College of Medicine, Southwest Jiaotong University, Chengdu, China.”

The original article has been updated.

