# Supplementary material for: Evaluating the impact of metabolic syndrome on aging in US adults: a cross-sectional study from NHANES
Source: Front Public Health. 2025 Jun 2;13:1593214. doi: 10.3389/fpubh.2025.1593214 (PMC12171360; doi:10.3389/fpubh.2025.1593214)
Supplement: Supplementary file 1 [file Table_1.pdf]

Supplement Table 1. The associations of metabolic syndrome with PhenoAgeAccel.

**Characteristic**	**Crude model**		**Model 1**		**Model 2**	
	**β (95 % CI)**	***P***	**β (95 % CI)**	***P***	**β (95 % CI)**	***P***
**Metabolic syndrome**						
No	ref		ref		ref	
Yes	3.59(3.13, 4.06)[a]	<0.0001	2.49(2.03, 2.95)[a]	<0.0001	0.61(0.12, 1.10)[a]	0.01
**Hypertension**						
No	ref		ref		ref	
Yes	2.11(1.62, 2.60)[a]	<0.0001	1.58(1.01, 2.15)[a]	<0.0001	0.92(0.36, 1.48)[a]	0.002
**Raised triglyceride**						
No	ref		ref		ref	
Yes	1.76(1.22, 2.30)[a]	<0.0001	0.94(0.45, 1.43)[a]	<0.001	0.15(-0.29, 0.60)	0.49
**Reduced HDL-C**						
No	ref		ref		ref	
Yes	2.21(1.74, 2.68)[a]	<0.0001	1.56(1.09, 2.02)[a]	<0.0001	0.66(0.28, 1.04)[a]	<0.001
**Central obesity**						
No	ref		ref		ref	
Yes	2.64(2.26, 3.02)[a]	<0.0001	0.65(0.24, 1.06)[a]	0.002	0.20(-0.17, 0.57)	0.29
**Raised blood glucose**						
No	ref		ref		ref	
Yes	3.70(3.25,4.16)[a]	<0.0001	3.14(2.71, 3.57)[a]	<0.0001	1.43(0.92,1.94) [a]	<0.0001

Abbreviations: CI, confidence interval;

Crude model: unadjusted;

Model 1: adjusted for age, sex, race, BMI;

Model 2: adjusted for variables in Model 1 plus, marital status, drinking status, smoking status, educational level, hypertension, diabetes, PIR, and HEI-2015.

[a] Statistically significant.

Supplement Table 2. The associations of metabolic syndrome with serum α-Klotho levels.

Characteristic	Crude model β (95 % CI)	*P*	Model 1 β (95 % CI)	*P*	Model 2 β (95 % CI)	*P*
**Metabolic syndrome**						
No	ref		ref		ref	
Yes	-30.39(-58.29,-2.49)[a]	0.03	-24.92(-45.67, -4.16)[a]	0.02	-37.94(-65.57,-10.30)[a]	0.01
**Hypertension**						
No	ref		ref		ref	
Yes	-16.01(-41.81,9.79)	0.22	-7.82(-32.92, 17.28)	0.53	-7.96(-37.18, 21.26)	0.55
**Raised triglyceride**						
No	ref		ref		ref	
Yes	-62.84(-99.37,-26.32)[a]	0.001	-56.37(-91.36,-21.38)[a]	0.003	-56.91(-97.34,-16.48)[a]	0.01
**Reduced HDL-C**						
No	ref		ref		ref	
Yes	-7.64(-41.45,26.18)	0.65	-9.37(-40.54, 21.81)	0.54	-15.44(-44.03, 13.14)	0.25
**Central obesity**						
No	ref		ref		ref	
Yes	-17.37(-49.20,14.45)	0.27	-25.38(-60.56, 9.80)	0.15	-22.5(-59.20, 14.20)	0.20
**Raised blood glucose**						
No	ref		ref		ref	
Yes	-11.61(-41.68,18.47)	0.44	5.72(-22.78, 34.22)	0.68	-1.42(-35.80, 32.95)	0.93

Abbreviations: CI, confidence interval;

Crude model: unadjusted;

Model 1: adjusted for age, sex, race, BMI;

Model 2: adjusted for variables in Model 1 plus, marital status, drinking status, smoking status, educational level, hypertension, diabetes, PIR, and HEI-2015.

[a] Statistically significant.

Supplement Table 3. The associations of metabolic syndrome with serum α-Klotho levels.

Characteristic	Crude model		Model 1		Model 2	
	β (95 % CI)	*P*	β (95 % CI)	*P*	β (95 % CI)	*P*
**Metabolic syndrome**						
No	ref		ref		ref	
Yes	-30.39(-58.29,-2.49)[a]	0.03	-24.92(-45.67, -4.16)[a]	0.02	-32.95(-62.91, -2.98)[a]	0.04
**Hypertension**						
No	ref		ref		ref	
Yes	-16.01(-41.81,9.79)	0.22	-7.82(-32.92, 17.28)	0.53	-1.79(-33.97, 30.38)	0.90
**Raised triglyceride**						
No	ref		ref		ref	
Yes	-62.84(-99.37,-26.32)[a]	0.001	-56.37(-91.36,-21.38)[a]	0.003	-51.95(-93.88,-10.01)[a]	0.02
**Reduced HDL-C**						
No	ref		ref		ref	
Yes	-7.64(-41.45,26.18)	0.65	-9.37(-40.54, 21.81)	0.54	-12.08(-42.39, 18.24)	0.38
**Central obesity**						
No	ref		ref		ref	
Yes	-17.37(-49.20,14.45)	0.27	-25.38(-60.56, 9.80)	0.15	-23.34(-62.06, 15.38)	0.20
**Raised blood glucose**						
No	ref		ref		ref	
Yes	-11.61(-41.68,18.47)	0.44	5.72(-22.78, 34.22)	0.68	-0.43(-35.22, 34.37)	0.98

Abbreviations: CI, confidence interval;
Crude model: unadjusted;
Model 1: adjusted for age, sex, race, BMI;
Model 2: adjusted for variables in Model 1 plus, marital status, drinking status, smoking status, educational level, hypertension, diabetes, PIR, eGFR, and HEI-2015.
a Statistically significant.

Supplement Table 4. The associations of metabolic syndrome with PhenoAge.

Characteristic	Crude model β (95 % CI)	*P*	Model 1 β (95 % CI)	*P*	Model 2 β (95 % CI)	*P*
**[b]Metabolic syndrome**						
No	ref		ref		ref	
Yes	14.87(13.96, 15.79)[a]	<0.0001	2.4(1.95,2.85)[a]	<0.0001	0.64(0.18, 1.10)[a]	0.01
**Hypertension**						
No	ref		ref		ref	
Yes	19.2(18.39,20.00)[a]	<0.0001	1.58(1.01,2.15)[a]	<0.0001	0.92(0.36, 1.48)[a]	0.002
**Raised triglyceride**						
No	ref		ref		ref	
Yes	6.56(5.50,7.63)[a]	<0.0001	0.94(0.45,1.43)[a]	<0.001	0.15(-0.29, 0.60)	0.49
**Reduced HDL-C**						
No	ref		ref		ref	
Yes	0.20(-0.72,1.12)	0.67	1.56(1.09, 2.02)[a]	<0.0001	0.66(0.28, 1.04)[a]	<0.001
**Central obesity**						
No	ref		ref		ref	
Yes	11.63(10.77, 12.50)[a]	<0.0001	0.24(-0.24,0.71)	0.32	0.39(-0.04, 0.82)	0.08
**Raised blood glucose**						
No	ref		ref		ref	
Yes	15.64(14.69, 16.58)[a]	<0.0001	3.13(2.70,3.55)[a]	<0.0001	1.27(0.75, 1.78)[a]	<0.0001

Abbreviations: CI, confidence interval;

Crude model: unadjusted;

Model 1: adjusted for age, sex, race, BMI;

Model 2: adjusted for variables in Model 1 plus, marital status, drinking status, smoking status, educational level, hypertension, diabetes, PIR, and HEI-2015.

[a] Statistically significant.

[b] Metabolic syndrome is defined according to the International Diabetes Federation

(IDF) - 2009 criteria.

Supplement Table 5. The associations of metabolic syndrome with PhenoAgeAccel.

Characteristic	Crude model		Model 1		Model 2	
	β (95 % CI)	*P*	β (95 % CI)	*P*	β (95 % CI)	*P*
**[b]Metabolic syndrome**						
No	ref		ref		ref	
Yes	3.37(2.95,3.80)[a]	<0.0001	2.40(1.95, 2.85)[a]	<0.0001	0.75(0.27, 1.24)[a]	0.003
**Hypertension**						
No	ref		ref		ref	
Yes	2.11(1.62,2.60)[a]	<0.0001	1.58(1.01, 2.15)[a]	<0.0001	0.92(0.36, 1.48)[a]	0.002
**Raised triglyceride**						
No	ref		ref		ref	
Yes	1.76(1.22,2.30)[a]	<0.0001	0.94(0.45,1.43)[a]	<0.001	0.15(-0.29, 0.60)	0.49
**Reduced HDL-C**						
No	ref		ref		ref	
Yes	2.21(1.74,2.68)	<0.0001	1.56(1.09, 2.02)[a]	<0.0001	0.66(0.28, 1.04)[a]	<0.001
**Central obesity**						
No	ref		ref		ref	
Yes	2.22(1.78,2.65)[a]	<0.0001	0.24(-0.24,0.71)	0.32	0.39(-0.04, 0.82)	0.08
**Raised blood glucose**						
No	ref		ref		ref	
Yes	3.67(3.22,4.13)[a]	<0.0001	3.13(2.70,3.55)[a]	<0.0001	1.27(0.75, 1.78)[a]	<0.0001

Abbreviations: CI, confidence interval;
Crude model: unadjusted;
Model 1: adjusted for age, sex, race, BMI;
Model 2: adjusted for variables in Model 1 plus, marital status, drinking status, smoking status, educational level, hypertension, diabetes, PIR, and HEI-2015.
[a] Statistically significant.
[b] Metabolic syndrome is defined according to the International Diabetes Federation (IDF) - 2009 criteria.

Supplement Table 6. The associations of metabolic syndrome with PhenoAge.

Characteristic	Crude model β (95 % CI)	*P*	Model 1 β (95 % CI)	*P*	Model 2 β (95 % CI)	*P*
**Metabolic syndrome**						
No	ref		ref		ref	
Yes	14.76(13.81,15.72)[a]	<0.0001	2.49(2.03, 2.95)[a]	<0.0001	0.64(0.15, 1.13)[a]	0.01
**Hypertension**						
No	ref		ref		ref	
Yes	19.2(18.39,20.00)[a]	<0.0001	1.58(1.01, 2.15)[a]	<0.0001	0.92(0.36, 1.46)[a]	0.001
**Raised triglyceride**						
No	ref		ref		ref	
Yes	6.56(5.50,7.63)[a]	<0.0001	0.94(0.45, 1.43)[a]	<0.001	0.18(-0.25, 0.60)	0.41
**Reduced HDL-C**						
No	ref		ref		ref	
Yes	0.20(-0.72,1.12)[a]	0.67	1.56(1.09, 2.02)[a]	<0.0001	0.81(0.45, 1.17)[a]	<0.0001
**Central obesity**						
No	ref		ref		ref	
Yes	10.61(9.71,11.51)[a]	<0.0001	0.65(0.24, 1.06)[a]	0.002	0.15(-0.22, 0.52)	0.42
**Raised blood glucose**						
No	ref		ref		ref	
Yes	15.54(14.60,16.48)[a]	<0.0001	3.14(2.71, 3.57)[a]	<0.0001	1.42(0.88, 1.97)[a]	<0.0001

Abbreviations: CI, confidence interval;

Crude model: unadjusted;

Model 1: adjusted for age, sex, race, BMI;

Model 2: adjusted for variables in Model 1 plus, marital status, drinking status, smoking status, educational level, hypertension, diabetes, PIR, year cycle, and HEI-2015.

[a] Statistically significant.

Supplement Table 7. The associations of metabolic syndrome with PhenoAgeAccel.

Characteristic	Crude model		Model 1		Model 2	
	β (95 % CI)	*P*	β (95 % CI)	*P*	β (95 % CI)	*P*
**Metabolic syndrome**						
No	ref		ref		ref	
Yes	3.59(3.13, 4.06)[a]	<0.0001	2.49(2.03, 2.95)[a]	<0.0001	0.64(0.15, 1.13)[a]	0.01
**Hypertension**						
No	ref		ref		ref	
Yes	2.11(1.62, 2.60)[a]	<0.0001	1.58(1.01, 2.15)[a]	<0.0001	0.92(0.38, 1.46)[a]	0.001
**Raised triglyceride**						
No	ref		ref		ref	
Yes	1.76(1.22, 2.30)[a]	<0.0001	0.94(0.45, 1.43)[a]	<0.001	0.18(-0.25, 0.60)	0.41
**Reduced HDL-C**						
No	ref		ref		ref	
Yes	2.21(1.74, 2.68)[a]	<0.0001	1.56(1.09, 2.02)[a]	<0.0001	0.81(0.45, 1.17)[a]	<0.0001
**Central obesity**						
No	ref		ref		ref	
Yes	2.64(2.26, 3.02)[a]	<0.0001	0.65(0.24, 1.06)[a]	0.002	0.15(-0.22, 0.52)	0.42
**Raised blood glucose**						
No	ref		ref		ref	
Yes	3.70(3.25,4.16)[a]	<0.0001	3.14(2.71, 3.57)[a]	<0.0001	1.42(0.88, 1.97) [a]	<0.0001

Abbreviations: CI, confidence interval;

Crude model: unadjusted;

Model 1: adjusted for age, sex, race, BMI;

Model 2: adjusted for variables in Model 1 plus, marital status, drinking status, smoking status, educational level, hypertension, diabetes, PIR, year cycle, and HEI-2015.

[a] Statistically significant.

Supplement Table 8. The associations of metabolic syndrome with PhenoAgeAccel in subgroups.

Characteristic	Metabolic Syndrome			
	No	Yes, β (95 % CI)[a]	*P*	*P* for interaction
**Sex**				0.06
Male	ref	1.01(0.35, 1.67)	0.003	
Female	ref	0.63(-0.01, 1.28)	0.05	
**Age group**				0.26
<65	ref	0.73(0.16, 1.31)	0.01	
>=65	ref	0.91(0.09, 1.73)	0.03	
**BMI group**				0.33
Overweight	ref	0.47(-0.16, 1.10)	0.14	
Obese	ref	0.93(0.27, 1.59)	0.01	
Normal	ref	1.26(-0.63, 3.14)	0.19	
**Race**				0.01
Non-Hispanic White	ref	0.96(0.38, 1.54)	0.002	
Non-Hispanic Black	ref	1.24(-0.17, 2.64)	0.08	
Mexican American	ref	0.13(-0.61, 0.88)	0.72	
Other	ref	-0.69(-2.17, 0.79)	0.35	
**PIR**				0.27
<1.3	ref	0.49(-0.58, 1.56)	0.36	
1.3-3.5	ref	0.75(-0.23, 1.74)	0.13	
≥3.5	ref	0.49(-0.58, 1.56)	0.36	
**Smoking status**				0.22
Former	ref	0.58(-0.11, 1.26)	0.10	
Never	ref	0.44(-0.08, 0.97)	0.10	
Now	ref	1.72(-0.09, 3.53)	0.06	
**Drinking status**				0.17
Former	ref	1.40(-0.17, 2.98)	0.08	
Heavy	ref	0.57(-0.78, 1.93)	0.40	
Moderate	ref	0.35(-0.65, 1.35)	0.49	
Mild	ref	0.43(-0.18, 1.04)	0.17	
Never	ref	1.11(-0.08, 2.31)	0.07	

Abbreviations: BMI, body mass index; CI, confidence interval; DM,diabetes; PreDM (IFG, impaired fasting glucose; IGT, impaired glucose golerance).

[a] Model adjusted for age, sex, race, BMI, marital status, drinking status, smoking status, educational level, hypertension, diabetes, PIR, and HEI-2015.
